# Supplementary material for: Identification of fibrillogenic regions in human triosephosphate isomerase
Source: PeerJ. 2016 Feb 4;4:e1676. doi: 10.7717/peerj.1676 (PMC4748702; doi:10.7717/peerj.1676)
Supplement: Table S1 [file peerj-04-1676-s002.docx]

| No | Name | Sequence | Region | Length (residues) | charge |
| --- | --- | --- | --- | --- | --- |
| 1 | CS-1 | EKVVFEQTKVIAD | 141-153 | 13 | -1 |
| 2 | CS-2 | VWAIGTGKT | 168-176 | 9 | 1 |
| 3 | CS-3 | PEFVDIINAKQ | 239-249 | 11 | -1 |
| 4 | β1 | KFFVGGNWK | 6-14 | 9 | 2 |
| 5 | β2 | DTEVVCAPP | 37-45 | 9 | -2 |
| 6 | β3 | KIAVAAQN | 59-66 | 8 | 1 |
| 7 | β4 | TWVVLGH | 90-96 | 7 | 1 |
| 8 | β5 | EGLGVIACIGE | 120-130 | 11 | -2 |
| 19 | β6 | KVVLAYEP | 160-167 | 8 | 0 |
| 10 | β7 | QSTRIIYGGSVT | 203-214 | 12 | 1 |
| 11 | β8 | DVDGFLVGGAS | 226-236 | 11 | -2 |

**Table S1.** Synthetic peptides tested in aggregation assays.
