# Supplementary figures and images for: Identification of fibrillogenic regions in human triosephosphate isomerase
Source: PeerJ. 2016 Feb 4;4:e1676. doi: 10.7717/peerj.1676 (PMC4748702; doi:10.7717/peerj.1676)

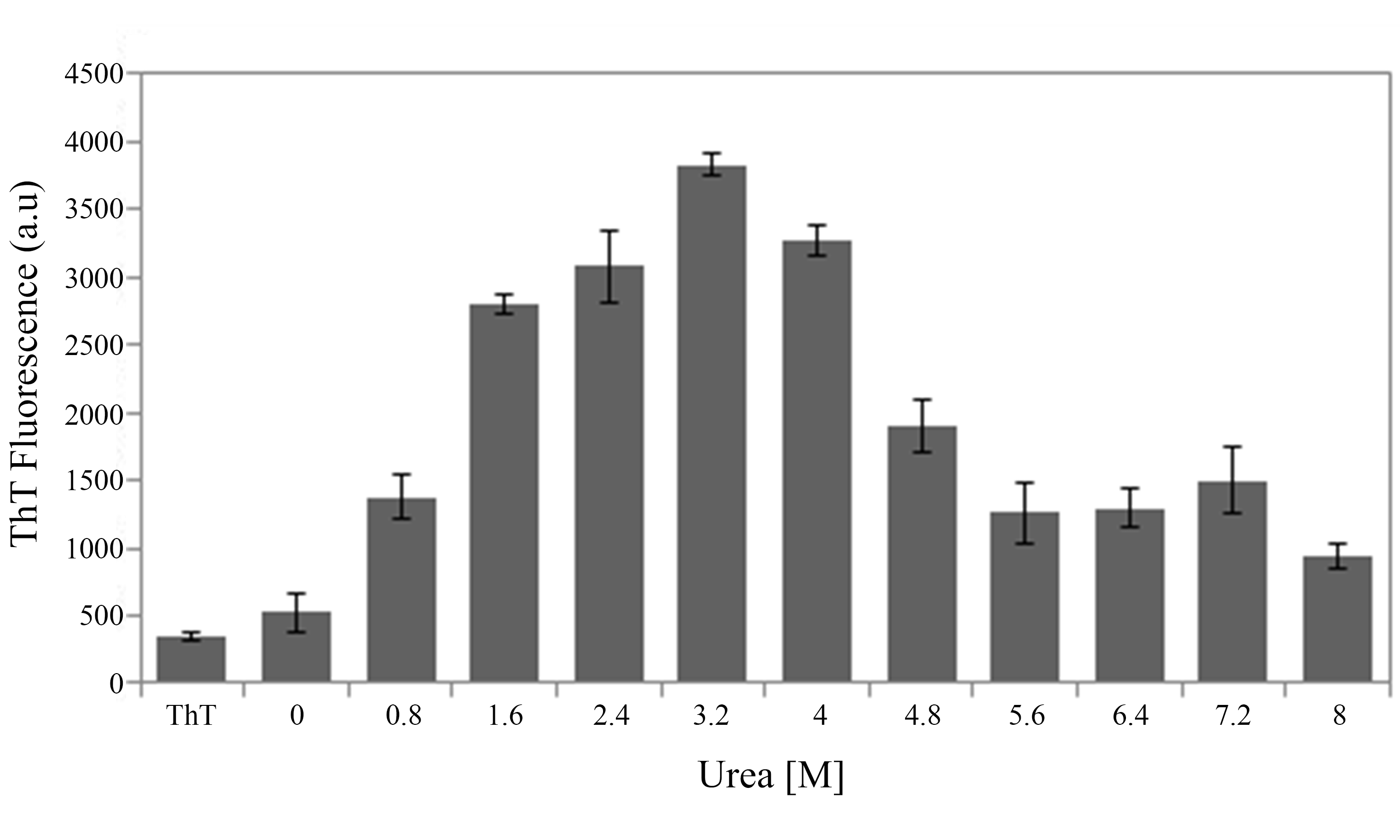

Supplement: Figure S2 [file peerj-04-1676-s003.png]
